# Selective constraints on protamine 2 in primates and rodents

**DOI:** 10.1186/s12862-016-0588-1

**Published:** 2016-01-22

**Authors:** Lena Lüke, Maximiliano Tourmente, Hernan Dopazo, François Serra, Eduardo R. S. Roldan

**Affiliations:** Reproductive Ecology and Biology Group, Museo Nacional de Ciencias Naturales (CSIC), c/Jose Gutierrez Abascal 2, 28006 Madrid, Spain; Department of Ecology, Genetics and Evolution, Universidad de Buenos Aires, Buenos Aires, Argentina; Centro Nacional de Análisis Genómico (CNAG-CRG), Center for Genomic Regulation, Universitat Pompeu Fabra, Barcelona, Spain

**Keywords:** Protamine, Evolution, Sexual selection, Sperm competition, Primates, Rodents, Spermatozoa

## Abstract

**Background:**

Protamines are sperm nuclear proteins with a crucial role in chromatin condensation. Their function is strongly linked to sperm head morphology and male fertility. Protamines appear to be affected by a complex pattern of selective constraints. Previous studies showed that sexual selection affects protamine coding sequence and expression in rodents. Here we analyze selective constraints and post-copulatory sexual selection acting on protamine 2 (*Prm2*) gene sequences of 53 species of primates and rodents. We focused on possible differences in selective constraints between these two clades and on the two functional domains of PRM2 (cleaved- and mature-PRM2). We also assessed if and how changes in *Prm2* coding sequence may affect sperm head dimensions.

**Results:**

The domain of *Prm2* that is cleaved off during binding to DNA (cleaved-*Prm2*) was found to be under purifying selection in both clades, whereas the domain that remains bound to DNA (mature-*Prm2*) was found to be positively selected in primates and under relaxed constraint in rodents. Changes in cleaved-*Prm2* coding sequence are significantly correlated to sperm head width and elongation in rodents. Contrary to expectations, a significant effect of sexual selection was not found on either domain or clade.

**Conclusions:**

Mature-PRM2 may be free to evolve under less constraint due to the existence of PRM1 as a more conserved and functionally redundant copy. The cleaved-PRM2 domain seems to play an important role in sperm head shaping. However, sexual selection on its sequence may be difficult to detect until it is identified which sperm head phenotype (shape and size) confers advantages for sperm performance in different mammalian clades.

**Electronic supplementary material:**

The online version of this article (doi:10.1186/s12862-016-0588-1) contains supplementary material, which is available to authorized users.

## Background

The evolution of reproductive phenotypes, and underlying selective forces, are the subject of much interest in evolutionary biology. Sperm competition, one of these selective forces, is known to affect sperm phenotype in males competing for the fertilization of ova [1] by driving adaptive changes of sperm morphology and function [2–4]. However, it is not yet clear how changes at the molecular level are linked to adaptations in sperm phenotype.

Responses to high levels of sperm competition include increases in sperm numbers, which are achieved by an increase in testes mass relative to body size [2, 4]. Relative testes mass is strongly associated to levels of sperm competition [2, 4, 5] and genetic paternity [6]. Thus, relative testes mass is widely used as proxy for level of sperm competition. Additional responses to high levels of sperm competition are increases in sperm swimming velocity, total sperm size and sperm quality (i.e., viability and morphology of sperm cells) [7–10]. The morphology of sperm cells, particularly the sperm head, varies considerably among species [11–13]. Sperm head dimensions, size of the apical hook, and head shape are also influenced by high levels of sperm competition [9, 14].

Studies on coding sequences of sperm proteins have identified proteins under the influence of sperm competition. The evolutionary rate of coding sequences of two seminal fluid proteins (SEMG2 and SVS), two sperm surface proteins (ADAM 2 and ADAM18), and proteins of the acrosome (Zonadhesin and SPAM1) seem to be positively related to level of sperm competition in primates [15–19]. Other studies found an increase of selective constraint in the presence of sperm competition. This effect was described for seminal fluid proteins in butterflies and sperm nuclear proteins protamine 1 (PRM1) and protamine 2 (PRM2) in rodents [20–22].

Protamines are small, arginine-rich sperm nuclear proteins. They are crucial for the condensation of sperm chromatin that takes place through successive protein replacements, first of histones by transition nuclear proteins, and then of the latter by protamines [23]. PRM1 is found throughout mammals, whereas PRM2 is found almost exclusively in primates and rodents. Evidence for the existence of PRM2 gene, transcripts and, in some cases, mature protein is available for a few other mammalian species [23–25]. *Prm2*, unlike *Prm1*, codes for a precursor, which is processed by successive proteolytic cleavages at the time of sperm differentiation [23, 24]. PRM2 processing occurs while DNA condensation is taking place and protamines are bound to DNA [26]. A mature form of PRM2 (hereafter, mature-PRM2) can be identified after cleavage. The role of the PRM2 domain that is cleaved off (hereafter, cleaved-PRM2) from the precursor is not clear. Cleaved-PRM2 and mature-PRM2 are structually and functionally different [21, 27]. The sequence of mature-*Prm2* resembles that of *Prm1*, which is consistent with the idea that *Prm2* has evolved as the result of *Prm1* gene duplication [21, 28]. Both *Prm1* and *Prm2* have DNA-anchoring domains containing 3–7 arginine residues separated by uncharged amino acids [25]. The arginine residues in protamines neutralize the charge of the DNA backbone and may also play a role in the activation of egg casein kinase II after fertilization [29].

Because of their important role during sperm chromatin condensation, alterations in protamine expression affect male fertility [24, 30–32]. In men, changes in sperm protamine content affect sperm head morphology and reduce sperm number and sperm motility [31]. Aberrant sperm chromatin condensation leads to larger and abnormal sperm heads [33]. In mice, an unbalanced protamine content associates with sperm DNA damage, sperm morphological abnormalities, and decreases in sperm motility [34]. Changes in protamine gene sequences and protamine expression ratios are linked to differences in head size and shape in muroid rodents [27, 35].

Protamines are thought to evolve fast, showing high structural heterogeneity [23, 36]. However, selective constraints are highly variable within the gene sequence and between taxa. Evidence of positive selection on the *Prm1* gene sequence has been detected in primates [36, 37] although the general trend for mammals is that the gene sequence is conserved [22]. Different selective constraints for *Prm1* and *Prm2* have been found in other mammalian species [21, 38]. Within mammals protamines are thought to be diverse, especially in the C-terminal region, but they contain conserved regions that are also found in birds (N-terminal ARYR, SRSRSR phosphorylation site, 3 arginine clusters) [39]. The high arginine content is thought to be conserved within the sequence, while the position of arginine residues seems to be highly variable [40]. A recent study found the high arginine content in *Prm1* to be driven by sexual selection in the form of sperm competition [22]. In a group of cricetid rodents, *Prm1* was shown to be under conserved selective constraint, with signs of positive selection restricted to specific codon sites. On the other hand, the two *Prm2* domains were shown to be under relaxed constraint on the way to degradation [21]. Sperm competition was shown to reduce the relaxation acting on the gene sequence of *Prm2*, resulting in a more conserved state of the gene in species with high levels of sperm competition [21].

In this study we examined the selective pressures potentially acting on *Prm2*. Since PRM2 is mainly expressed in rodents and primates this study concentrated on these clades. In addition, because the PRM2 precursor actually contains two structurally and functionally different domains (cleaved-PRM2 and mature-PRM2) we analyzed them separately to examine the possibility that they may be under different selective pressures. Further to a comparison of selective pressures, we examined the possible effects of postcopulatory sexual selection (sperm competition) on the coding sequence. Since sexual selection has been shown to affect arginine content in *Prm1* we also tested for effects of postcopulatory sexual selection on arginine content of mature-*Prm2*. We predicted that differences could exist in selective constraints on *Prm2* between primates and rodents. Since sexual selection was already shown to affect *Prm2* in cricetid rodents we anticipated signs of sexual selection for all rodents. Finally, we predicted that cleaved-*Prm2* and mature-*Prm2* could evolve under different selective regimes.

## Results

### Sequence properties

Coding sequences and arginine contents were compared between primates and rodents (Additional file 1: Table S1). Cleaved-*Prm2* sequence was significantly longer in primates (t_*24.08*_ = 7.22, *P* <0.001) whereas mature-*Prm2* was significantly longer in rodents (t_*20.5*_ = −13.5, *P* <0.001). No significant difference was found in mature-*Prm2* arginine content between primates and rodents (t_*22.87*_ = −0.13, *P* = 0.9).

### Selective pressures across species

We tested for the general trend of selection acting on *Prm2* domains across all species. Mammalian species other than rodents and primates were included to provide a background for comparisons. To obtain the background pressure acting on the whole sequence across all species we calculated the evolutionary rate (ω) (see Methods: “Analysis of selective pressures”) for the whole tree on the entire sequence (Codeml PAML4 model M0 as explained in Methods). The evolutionary rate calculated across all species in model M0 for cleaved-*Prm2* was ω = 0.54, and for mature-*Prm2* it was ω = 1.18.

### Comparison of selective pressures

To compare selective pressures for the whole sequence and selective pressure on codon sites we used a branch analysis and a branch-site analysis. In each analysis we first marked primates as foreground against the other species as background, and then marked rodents as foreground against the other species as background (see Methods: “Analysis of selective pressures”).

These evolutionary “clade” models (MC), constraining the evolutionary rates of cleaved-*Prm2* in either primates or rodents showed no differences between clades. Selective pressures did not differ significantly from the background for the two clades (primates and rodents: M0 vs MC not significant, M0 ω = 0.54). The calculated evolutionary rate of cleaved-*Prm2* was significantly different from 1 for both primates and rodents (MCfix vs MC significant, M0 ω = 0.54); the low evolutionary rate suggests that the domain is under weak purifying selection in both clades.

This same branch analysis on mature-*Prm2* revealed that primates had significantly lower selective constraints than rodents (primates: M0 vs MC significant, MC ω = 3.12) and that rodents did not evolve with a rate that was significantly different from that of the background (rodents: M0 vs MC not significant, M0 ω = 1.18) (Table 1). These results suggest positive selection for mature-*Prm2* in primates. An evolutionary model allowing an excess of non-synonymous mutations was significantly more likely than a completely neutral evolutionary model (see significant differences between MC and MCfix in Table 1). For mature-*Prm2* of rodents, neutral evolutionary models were the most likely (see MC vs MCfix in Table 1).

**Table 1:** Summary of results for branch analysis and branch-site analysis of *Prm2* domains of primates and rodents

Sequence	cleaved *Prm2*		mature *Prm2*	
Foreground	Primates	Rodentia	Primates	Rodentia
LRTs for selection at branches over whole sequence				
2Δ(M0-MC)	0.02	1.94	25.86	1.49
p	ns	ns	<0.01	ns
2Δ(MCfix-MC)	8.74	15.42	25.60	0.01
p	0.01	<0.01	<0.01	ns
M0 - ω	0.54		1.18	
MC - ω	0.530	0.420	3.120	0.980
LRTs for selection at branches on sites				
2Δ(M1-BS)	1.67	5.27	62.21	26.10
p	ns	ns	<0.01	<0.01
2Δ(BSfix-BS)	0.00	5.27	96.75	60.63
p	ns	0.05	<0.01	<0.01
Proportion of sites in ω site classes				
0	0.23	0.23	0.18	0.21
1	0.64	0.74	0.30	0.75
2a	0.04	0.01	0.20	0.01
2b	0.10	0.02	0.32	0.03
Positively selected sites (BEB *p* <0.05)				
PSS	-	26G	1Q, 4C, 5Y, 6G, 7Y, 11 L, 24Q, 25R, 29R, 44R, 45 N, 51R, 55 T, 61 T	64R,72H
Interpretation				
Selection at branches over whole sequence	conserved	conserved	positive	relaxed
Selection at branches on sites	no signal	positive	positive	positive
Sexual selection	not detected	not detected	not detected	not detected

LRT: Likelihood ratio test (twice the difference (2Δ) between likelihood values of the tested models). ω: nonsynonymous/synonymous substitution rate ratio, evolutionary rate. When LRT of M0 versus MC is significant MC omega is reported. When LRT is non significant, M0 omega is reported

PSS: positively selected sites. Ω site classes: 0: 0 < ω < 1 for foreground and background branches, 1: ω = 1 for foreground and background branches, 2a: 0 < ω < 1 for background and ω > 1 for foreground, 2b: ω = 1 for background and ω > 1 for foreground

Although the branch tests we performed are adequate for detecting and comparing global evolutionary trends, these models are blind to positive selection or relaxation on specific sites. To address the latter we used the so-called branch-site test (see Methods).

**Fig. 1:**
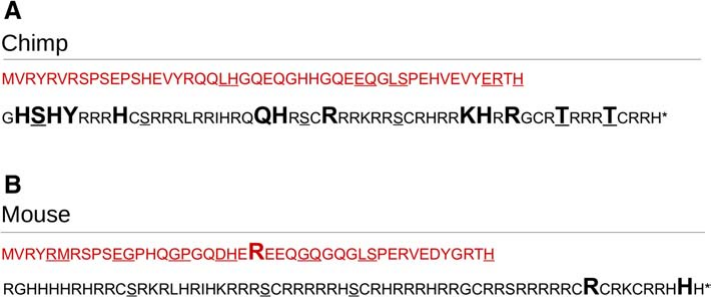
Representation of positively selected sites (PSS) for **a** primates, visualized on the PRM2 amino acid sequence of *Pan troglodytes* (Chimp) and **b** rodents, visualized on the PRM2 amino acid sequence of *Mus musculus musculus* (Mouse). Cleaved*-*PRM2 is shown in red. PSS as detected by branch-site analysis (see Methods) are enlarged and boldface. Post-translational processing (cleavage) sites are underlined in cleaved*-*PRM2 [25, 105]. Proposed phosphorylation sites [25] are underlined in mature*-*PRM2

The branch-site test revealed no directed selection on codon sites for cleaved-*Prm2* in primates (BSfixed vs BS non significant), while for rodents one codon site of cleaved-*Prm2* was shown to be positively selected (BSfixed vs BS significant) (Table 1, Fig. 1). For mature-*Prm2*, both primates and rodents showed significantly positively selected codon sites within the alignment (BSfixed vs BS significant) (Table 1, Fig. 1).

The root-to-tip ω calculated for all species is shown in Additional file 1: Table S1.

### Sexual selection

To test for sexual selection on *Prm2* coding sequences in primates and rodents we chose the phylogenetic generalized least squares (PGLS) regression analysis (see Methods). The root-to-tip ω, as well as and arginine content (as percent of sequence length), were included as dependent variables against the independent variables body mass and testes mass (i.e., relative testes mass, which serves as proxy for sperm competition). In addition, we tested for an effect of evolutionary rate on arginine content.

No significant correlations were found between residual testes mass and root-to-tip ω values of either cleaved- or mature-*Prm2* domains or between residual testes mass and arginine content of mature-*Prm2* in primates or rodents. Arginine content was not correlated with mature-*Prm2* root-to-tip ω (Table 2).

**Table 2:** Phylogenetically-controlled regression analyses

Clade	Dependent value	Independent value	n	Slope	t	R2	λ	*p*
cleaved-*Prm2*								
Primates	cleaved-*Prm2* ω	log body mass	12	0.02	0.23	0.14	1(ns,ns)	0.82
		log testes mass		0.60	0.83			0.43
Rodents	cleaved-*Prm2* ω	log body mass	28	0.00	0.27	0.01	1(ns,ns)	0.79
		log testes mass		0.00	−0.35			0.73
Primates	relative head length	cleaved-*Prm2* ω	11	0.02	0.07	0.00	1(ns,ns)	0.94
Rodents	relative head length	cleaved-*Prm2* ω	26	0.18	1.83	0.12	0.41(ns,ns)	0.08
**Rodents**	**relative head width**	**cleaved-** ***Prm2*** **ω**	**22**	**0.14**	**2.33**	**0.20**	0.91(*,ns)	**0.03**
**Rodents**	**sperm head elongation**	**cleaved-** ***Prm2*** **ω**	**22**	**−8.07**	**−3.11**	**0.32**	0.91(*,ns)	**0.00**
mature-*Prm2*								
Primates	mature-*Prm2* ω	log body mass	12	0.02	1.19	0.27	0.86(ns,ns)	0.26
		log testes mass		0.00	0.36			0.73
Rodents	mature-*Prm2* ω	log body mass	28	−0.03	−1.80	0.17	1(*,ns)	0.09
		log testes mass		−0.01	−0.30			0.77
Primates	Arginine content (mature-*Prm2*)	log body mass	12	−1.32	−1.49	0.21	1(*,ns)	0.17
		log testes mass		0.47	0.48			0.65
Rodents	Arginine content (mature-*Prm2*)	log body mass	28	−0.02	−0.36	0.03	1(*,ns)	0.72
		log testes mass		−0.02	−0.52			0.61
Primates	relative head length	mature-*Prm2* ω	10	−0.02	−0.82	0.07	1(*,ns)	0.43
Rodents	relative head length	mature-*Prm2* ω	26	0.03	1.15	0.05	0(ns,ns)	0.26
Rodents	relative head width	mature-*Prm2* ω	22	0.03	0.94	0.04	0.96(*,ns)	0.36
Rodents	sperm head elongation	mature-*Prm2* ω	22	−1.08	−0.83	0.03	1(*,ns)	0.41

The superscripts following the λ value indicate significance levels (ns: *p* >0.05; *: *p* <0.05) in likelihood ratio tests against models with λ = 0 (first superscript) and λ = 1 (second superscript). Abbreviations: n: number of species in analysis. Significant regression results are shown in boldface

### Relationships with sperm head dimensions

**Fig. 2:**
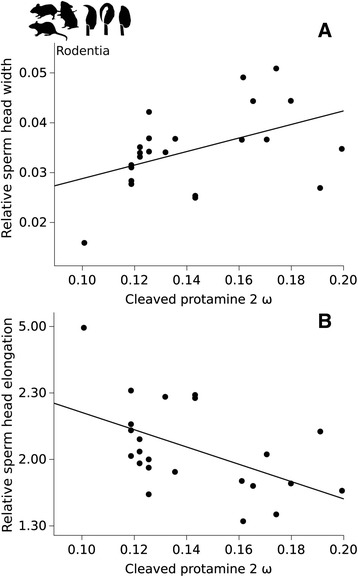
Visualization of significant PGLS regression results for **a** Relationship in rodents between cleaved*-Prm2* ω (root-to-tip ω) with sperm head width (relative to total sperm length) and **b** Relationship in rodents between cleaved*-Prm2* ω (root-to-tip ω) with sperm head elongation (sperm head length divided by sperm head width)

We tested for possible relationships between changes in the coding sequence of *Prm2* domains and sperm head dimensions. The evolutionary rate was used as independent variable in PGLS analyses, with relative head length (HL), relative head width (HW), and head elongation (HL/HW) used as dependent variables. For primates, data available for relative HW were not sufficient for regression analysis. PGLS regressions showed no significant correlations with relative HL or head elongation in primates (Table 2). In rodents, PGLS regressions showed a significant positive correlation between cleaved-*Prm2* root-to-tip ω and relative HW, and a significant negative correlation between cleaved-*Prm2* root-to-tip ω and head elongation (Fig. 2, Table 2). No significant correlations were found for mature-*Prm2*.

## Discussion

In this comparative study, focusing on possible selective constraints acting on the *Prm2* gene, we were able to demonstrate significant differences between evolutionary rates of primate and rodent *Prm2* as well as between cleaved- and mature-*Prm2* domains. In primates and rodents, cleaved-*Prm2* is conserved although one site was found to be positively selected in rodents. Mature-*Prm2* is under relaxed constraint in rodents and positively selected in primates. Additionally, we found directed positive selection on specific codon sites of mature-*Prm2* in both primates and rodents. A previous study concentrating solely on the cricetid family of rodents [27] showed how changes in cleaved-*Prm2* gene sequence associates with sperm head width and elongation. Here we present evidence for this relationship to be true across rodents. No signal of sexual selection was found for primates or rodents.

### Differences in selective constraints between taxa

Studies in rodents demonstrated that protamine gene sequences and protamine expression ratios influence sperm head size and shape [22, 27, 35]. The gene sequence of *Prm1* seems to be highly variable in mammals although highly conserved regions can also be identified [36, 40]. *Prm1* seems to have an unusual form of evolution which seems to be driven by sexual selection [22, 23, 40]. This complex pattern of selective constraints and sexual selection could be a consequence of the importance of PRM1 for sperm form and function resulting in a delicate balance between conservation of function and adaptations to high sperm competition levels. We expected to find an even more complex pattern of evolution in *Prm2* due to the existence of two domains in this protein. We were able to show differences in selective constraints between primates and rodents, especially for mature-*Prm2*, which is positively selected in primates and is under relaxed constraint in rodents. Cleaved-*Prm2* is conserved in both clades. This result is important in connection to the proposed functional redundancy of PRM1 and mature-PRM2. Mature-*Prm2* is thought to be the result of *Prm1* gene duplication [21, 28] and despite the proposed slight differences in function, mainly associated to the process of DNA condensation [27], the function of mature-PRM2 is essentially redundant to that of PRM1. This might be an explanation for the comparative lack of selective constraint of mature-*Prm2*. Due to the existence of two protamines, one may be “free” to evolve adaptively or under relaxed constraint while the other is more conserved with a more directed pattern of positive selection on specific codon sites to ensure proper function [21] (Table 3). When comparing selective constraints between primates and rodents differences in effective population sizes need to be taken into account because population size is generally lower in primates. Genetic drift can therefore be an explanation for the higher mature-*Prm2* evolutionary rate in primates [41]. The sequence might thus not be truly positively selected but, rather, may be under relaxed constraint with an effect increased by genetic drift. However, the general trend towards a lower selective constraint in mature-*Prm2* is clear.

**Table 3:** Comparison of selective constraints in mature*-Prm2* and *Prm1* of rodents and primates

Clade	mature-*Prm2*	*Prm1*
Primates	positive selection	relaxed constraint
Rodents	relaxed constraint	purifying selection

Results for *Prm1* are from Lüke et al. [22]

### No sexual selection detected for rodents or primates

We did not find signs of sexual selection acting on *Prm2* domains of primates or rodents. In a previous study, postcopulatory sexual selection was found to halt the relaxation in *Prm2* of cricetid rodents [21]. Here, the joint analysis of murids and cricetids did not show such relationship.

It has been proposed that the effect of sexual selection on protamines may result in modifications of the shape of the sperm head. High levels of sperm competition could promote changes in the sperm head that would render it more hydrodynamically efficient which, in turn, would influence sperm velocity. So far, it is not clear how changes in sperm head shape affect sperm velocity. Given the considerable variation in sperm head shape and size [11–13], the diversity in flagellar beating patterns, and the environments present in the female tract in mammals, it is fair to assume that adaptations of sperm head shape would be the result of a complex interplay between these factors [9, 42, 43]. Wider sperm heads, for example, might be advantageous for certain sperm morphologies whereas narrower heads may be more adpative for other sperm morphs. The effects of sexual selection may therefore be variable, and even contradictory, between different groups of species. Evidence for a complex pattern of selective pressures has been shown for ADAM proteins. In these sperm proteins positive selection within the adhesion domain has been attributed to adaptations to sperm competition and fertilization environment in primates, while in mouse species positive selection could not be explained by sexual selection [44]. A study comparing groups of species at deeper taxonomic levels, and including more species, might shed more light on the role of sperm competition on *Prm2* evolution.

Like PRM1, mature-PRM2 is very rich in arginine. The DNA-anchoring domains contain 3–7 arginine residues separated by uncharged amino acids [25]. Arginine neutralizes the charge of the DNA backbone and may play a role after fertilization [29]. For *Prm1*, sexual selection seems to be targeting especially the arginine coding content of the gene. Sperm competition seems to maintain high arginine content of PRM1 through sequence conservation. Species experiencing higher selective pressure through sperm competition show higher arginine content in the PRM1 amino acid sequence [22]. Unlike what was observed for *Prm1*, we did not find a relationship between sexual selection and arginine content in mature-PRM2. This might be explained by the fact that the arginine content of mature-PRM2 seems to be stable across primates and rodents, showing very low variability, leading to the conclusion that it is highly conserved.

### Positive selection on functionally important sites

Positively selected codon sites were found in both primates and rodents. In primates this positive selection is entirely concentrated on mature-*Prm2* codon sites. We found 13 postively selected sites in primate mature-*Prm2*. Interestingly 6 of them fall directly on or around proposed phosphorylation sites in the C- and N-terminal regions. Protamine phosphorylation is crucial for the DNA condensation process but the mechanism by which it affects DNA condensation is not known. It has been proposed that the phosphorylation of protamines is required for DNA binding while its subsequent dephosphorylation might be important in correct chromatin compaction [23, 45]. Changes in phosphorylation sites might affect the degree and efficiency of DNA condensation. If primate mature-PRM2 three-dimensional structure and binding mechanism resembles the proposed DNA binding model of PRM1 [46], changes in phosphorylation of the C- and N-terminal regions might affect DNA binding mechanism and cross-linking of protamines [46, 47].

In mice we found three positively selected sites, one in the cleaved-*Prm2* sequence and two in the C-terminal part of mature-*Prm2*. Unlike the situation in primates, these sites are not concentrated around proposed phosphorylation sites. However, since theses sites are positively selected they are likely to be of functional importance.

It is possible that accelerated evolution of these codon sites is an adaptation to selective pressures due to sperm competition. In order to understand how the rapid evolution of these sites affects protamine function, a comparative study including data on sequence evolution, sperm competition level, protamine phosphorylation and degree of chromatin compaction should be carried out in the future.

### Cleaved protamine 2

Our results show that cleaved-*Prm2* is conserved in rodents and primates, although we found one codon site to be positively selected in rodent cleaved-*Prm2*. In agreement with previous studies, we found that changes in the cleaved-*Prm2* coding sequence associate with wider and more elongated sperm heads in rodents [27]. The role of cleaved-PRM2 is not yet clear but its conservation and the apparent influence it has on sperm head shape speaks for an important function especially in relation to sperm competitiveness. The unprocessed PRM2 precursor binds to DNA and, while bound, is cleaved over a period of several days until only mature-PRM2 is left bound to DNA [48, 49]. Sperm chromatin condensation was shown to coincide temporally with the start of protamine translation and posttranslational processing [26, 50]. Therefore, it was proposed that the cleaved-PRM2 domain may have a more important role during the actual process of chromatin condensation than mature-PRM2 [27]. In order to understand its role in sperm competitiveness and male fertility the function of cleaved-PRM2 should be studied in more detail.

## Conclusions

As predicted, we found significant differences in selective constraints of the two *Prm2* domains (cleaved- and mature-*Prm2*) as well as differences between the two clades studied (primates and rodents). Mature-*Prm2* is generally relaxed in rodents with directed positive selection on sites and positively selected in primates. Mature-*Prm2* exhibits less constraint than its functionally redundant partner *Prm1*. We propose that mature-*Prm2* is free to evolve adaptively, or under less constraint, due to the existence of a more conserved, functional copy with redundant functional properties. Positive selection on codon sites is concentrated on primate mature-*Prm2* targeting possible phosphorylation sites and thus possibly affecting protamine function and chromatin condesation.

On the other hand, cleaved-*Prm2* is conserved in both clades with signs of positive selection on codon sites in rodents. We were also able to demonstrate that changes in cleaved-*Prm2* affect sperm head width and elongation across rodents. This domain seems to play an important role in the process of sperm chromatin condensation and sperm head shaping. Further studies should focus on the function of this important PRM2 domain. The fact that sexual selection was not detected in rodents or primates might be the result of differential interactions or trade-offs between sperm traits and its environment. A comparative study including a broader range of species might explain the complex patterns of sexual selection of *Prm2*.

## Methods

### Ethics statement

No research on live animals was conducted in this study. Our work is based on data available from public sources.

### Sequence data and phylogenetic tree

*Prm2* gene sequences of primate and rodent species, as well as those available for other mammalian species, were taken from NCBI Genbank and previous publications, all publicly available sources [9, 11, 17, 21, 27, 51–61] (Additional file 1: Table S1). Codon based alignments were performed using the muscle alignment algorithm implemented in Geneious 5.5.9. Arginine frequencies were calculated using Geneious 5.5.9 (Additional file 1: Table S1). The phylogenetic tree of the 53 mammalian species included in this study was constructed as a consensus of phylogenies available in the literature [62–91] (Additional file 2: Figure S1).

### Phenotype data

Data on body mass, testes mass and sperm dimensions were obtained from the literature; i.e., from publicly available sources [9, 11, 17, 27, 51–61] (see Additional file 1: Table S1). Testes and body mass data were available for 46 of the 53 species for which sequence data were available. Data on sperm head width were available for 30 species and sperm head length for 44 species. Residual testes mass data were obtained from a regression analysis including body mass as independent variable and testes mass as dependent variable. Residual testes mass was only used for graphical representation of multiple regression results. Because total sperm length varies greatly among these species, and drag resulting from head size should be analyzed taking into account the length of the flagellum [27, 92], sperm head length (HL) and head width (HW) were each used as proportion of total sperm length (hereafter, relative HL and relative HW).

### Analysis of selective pressures

The nonsynonymous/synonymous substitutions rate ratio (ω = dN/dS) is an indicator of selective pressure at the protein level, with ω = 1 indicating neutral evolution, ω < 1 purifying selection, and ω > 1 diversifying positive selection [93]. To estimate rates of sequence evolution we used the application codeml implemented in PAML 4 [94, 95]. Likelihood-ratio-tests (LRT) were performed to test if the alternative model presents a better fit to the dataset against the null model. For the codeml codon frequency setting, as well as the setting for number of categories, we used the setting with the best fit for each analysis according to the preliminary likelihood-ratio-analysis. Branch lengths calculated in the model M0 “one-ratio” (see below) where used as input for subsequent models.

#### Evolutionary rate (root-to-tip ω)

We used the free ratio model in Codeml (PAML4) in order to obtain species-specific ω values. The free ratio model calculates ω freely for each branch in the tree. Species root-to-tip ω was subsequently calculated by addition of dN values and dS values from the root of the clade to the terminal species branch of the respective clade and taking the ratio of the sum to obtain the root-to-tip ω value [21, 96].

#### Branch analysis

In order to obtain the evolutionary rate of clades and groups of species we performed a branch analysis comparing marked foreground branches against the unmarked background in the phylogenetic tree. For our analysis we marked either primate or rodent branches as foreground. All branches belonging to the respective species group are marked up to, and including, the last common ancestor of the group. Three models were computed: M0 “one ratio” in which all branches were constrained to evolve at the same rate; MCfixed “two-ratio, foreground fixed” where the background branches ω were allowed to be estimated freely while the foreground ω was restrained to a value of ω = 1; and MC “two ratio” model which estimates for both background and foreground branches a free and independent ω. To test if the foreground evolves at a significantly different rate than the background we compared M0 versus MC by means of LRT. If the foreground ω was significantly higher than 1 (LRT significant for MCfixed vs MC and ω > 1) we assumed positive selection acting on the foreground branches at whole sequence level. If the foreground ω was significantly lower than 1 (LRT significant for MCfixed vs MC and ω > 1) we report purifying selection acting on the branch at whole sequence level. Relaxed selective constraint for the foreground branch is assumed if the foreground evolves at a significantly different ω than the background (M0 vs MC), and this ω was not significantly different from 1 (MCfixed vs MC) [97]. See Additional file 3: Figure S2 for a graphical representation of the analysis.

#### Branch-site analysis

The branch analysis described above is used for the detection of general trends of selection on the whole gene sequence. However this test is not able to detect positive selection, or relaxation, on specific codon sites. For this aim we performed the so-called branch-site test. We computed two models to test evolution among coding sequences and infer amino acids under positive selection for marked foreground branches in contrast to the unmarked background. BSfixed “branch-site model A, foreground fixed” in which the codon site ω for background branches is allowed to be computed freely and BS “branch-site model A” in which codon sites in both foreground and background were computed freely [98]. Evidence of the existence of positively selected codon sites (PSS) is reported if LRT between BSfixed and BS is significant and sites significantly belonging to the positive selected site category are reported by the model.

### Phyologenetically corrected regression analysis (PGLS)

To test for correlations between variables we employed the phylogenetic generalized least squares approach (PGLS) [99]. Body mass and testes mass were included as independent variables in a multiple PGLS regression as a proxy for sperm competition (hereafter: relative testes mass). Analyses of associations between genetic and morphometric traits also took into account that such traits are not independent from their phylogenetic history [100]. The PGLS approach has been shown to be a powerful tool to detect associations of this kind [100], and it has been used in earlier studies in combination with the root-to tip dN/dS method showing genetic-morphometric associations [21, 96, 102, 103]. We performed PGLS analysis using CAPER v0.5 [104] package for R (v3.0.1; R Foundation for Statistical Computing 2013).

### Availability of supporting data

Gene sequences are available from NCBI Genbank and earlier publications (see details in Additional file 1: Table S1). Phenotypic data (body mass, testes mass and sperm dimensions) were available from the literature (compiled in Additional file 1: Table S1).

## Additional files

Additional file 1: Table S1. Data included in study. (PDF 54 kb) Additional file 2: Figure S1. Phylogenetic tree constructed as consensus of phylogenetic data available in the literature. (PDF 650 kb) Additional file 3: Figure S2. Graphical representation of the analyses carried out in this study. (PDF 1607 kb)

## Abbreviations

Cleaved-*Prm2*: sequence coding for protamine 2 domain cleaved off during post-translational processing
Cleaved-PRM*2*: Protamine 2 domain cleaved off during post-translational processing
HL: sperm head length
HW: sperm head width
LRT: Likelihood ratio test
Mature-PRM2: Protamine 2 domain remaining after post-translational processing
Mature-*Prm2*: sequence coding for protamine 2 domain remaining after post-translational processing
PGLS: Phylogenetic generalized least squares
*Prm2*: Protamine 2 coding sequence
PRM2: Protamine 2
PSS: positively selected sites
