# Supplementary material for: Selective constraints on protamine 2 in primates and rodents
Source: BMC Evol Biol. 2016 Jan 22;16:21. doi: 10.1186/s12862-016-0588-1 (PMC4724148; doi:10.1186/s12862-016-0588-1)
Supplement: Additional file 1: Table S1. — Data included in study. (PDF 54 kb) [file 12862_2016_588_MOESM1_ESM.pdf]

Table S1: Data included in study. HW= sperm head width, HL= sperm head length, TSL= total sperm length, BMASS= body mass, TMASS= testes mass

taxa	Order	Family	mature-Prm2 ω	cleaved-Prm2 ω	% arginine in mature-Prm2	HW/TSL	HL/TSL	HL/HW	BMASS (g, log)	TMASS (g, log)	Length cleaved-Prm2	Length mature-Prm2	Accession Prm2	References sperm dimensions	References body mass and testes mass
Alouatta_seniculus	Primates	Atelidae	1.38	0.26	50.00						48.00	52.00	X71335		
Apodemus_sylvaticus	Rodentia	Muridae	0.71	0.13	49.21	0.04	0.07	1.74	1.48	-0.02	44.00	63.00	FJ411393	Gómez Montoto et al 2011b	Gómez Montoto et al 2011a
Arvicola_sapidus	Rodentia	Cricetidae	0.50	0.20	55.74	0.03	0.06	1.76	2.34	0.35	44.00	61.00	Lüke et al 2011	Lüke et al 2014	Gómez Montoto et al 2011
Arvicola_terrestris	Rodentia	Cricetidae	0.50	0.18	55.74				1.96	-0.39	44.00	61.00	Lüke et al 2011	Gómez Montoto et al 2011b	Lüke et al 2014
Bos_taurus	Artiodactyla	Bovidae	1.59	0.28	60.42	0.08	0.13	1.57	5.83	2.83	46.00	48.00	BK006493	Cummins & Woodall 1985; Gage 1998	Kenagy & Trombulak 1986
Callithrix_jacchus	Primates	Callithricidae	1.14	0.21	58.93		0.11		2.51	0.11	48.00	56.00	X85371	Cummins & Woodall 1985	Kenagy & Trombulak 1986
Canis_familiaris	Carnivora	Canidae	0.93	0.33	54.55	0.08	0.10	1.30	4.33	1.44	45.00	55.00	BK006497	Anderson et al 2005; Steklenev 1975	Woodall & Johnsten 1988
Chionomys_nivalis	Rodentia	Cricetidae	0.54	0.17	55.00	0.04	0.08	1.80	1.69	-0.01	44.00	60.00	Lüke et al 2011	Gómez Montoto et al 2011b	Gómez Montoto et al 2011a
Clethrionomys_glareolus	Rodentia	Cricetidae	0.61	0.18	55.74	0.04	0.08	1.82	1.40	-0.37	44.00	61.00	Lüke et al 2011	Gómez Montoto et al 2011b	Gómez Montoto et al 2011a
Echinops_telfairi	Afrosoricida	Tenrecidae									48.00	58.00	BK006491		
Equus_caballus	Perissodactyla	Equidae	1.30	0.34	61.40	0.05	0.12	2.12	5.67	2.62	44.00	57.00	BK006494	Cummins & Woodall 1985; Gage 1998	Kenagy & Trombulak 1986
Erythrocebus_patas	Primates	Cercopithecidae	1.16	0.26	54.55		0.08		4.11	0.86	48.00	55.00	AF195644	Anderson et al 2005	Dixson & Anderson 2004
Felis_catus	Carnivora	Felidae	1.27	0.45	53.19	0.04	0.09	2.05	3.49	0.37	45.00	47.00	BK006496	Terrell et al 2011	França & Godinho 2003
Gorilla_gorilla	Primates	Pongidae	1.76	0.31	48.15		0.08		5.13	1.37	48.00	54.00	X71336	Cummins & Woodall 1985	Kenagy & Trombulak 1986
Homo_sapiens	Primates	Hominidae	1.88	0.27	48.15		0.08		4.80	1.70	48.00	54.00	AF215713	Anderson et al 2005	Kenagy & Trombulak 1986
Hylobates_lar	Primates	Hylobatidae	1.61	0.29	50.00		0.11		3.74	0.74	48.00	54.00	X71339	Anderson et al 2005	Kenagy & Trombulak 1986
Macaca_fuscata	Primates	Cercopithecidae	1.32	0.27	58.18						48.00	55.00	AB101300		
Macaca_mulatta	Primates	Cercopithecidae	1.43	0.26	55.56	0.04	0.07	1.66	4.02	1.88	48.00	54.00	X71338	Anderson et al 2005; Cummins & Woodall 1985	Kenagy & Trombulak 1986
Macaca_nemestrina	Primates	Cercopithecidae	1.32	0.27	58.18		0.08		4.00	1.82	48.00	55.00	X71340	Anderson et al 2005	Kenagy & Trombulak 1986
Mesocricetus_auratus	Rodentia	Cricetidae	0.56	0.10	54.84	0.02	0.05	2.99	2.10	0.54	44.00	62.00	AF268204.1	Gage & Freckleton 2003	Kenagy & Trombulak 1986
Microtus_agrestis	Rodentia	Cricetidae	0.66	0.17	57.38		0.07		1.67	-0.10	44.00	61.00	Lüke et al 2011	Cummins & Woodall 1985	Kenagy & Trombulak 1986
Microtus_arvalis	Rodentia	Cricetidae	0.67	0.17	56.45	0.04	0.07	2.04	1.66	-0.32	42.00	62.00	Lüke et al 2011	Gómez Montoto et al 2011b	Gómez Montoto et al 2011a
Microtus_cabrerae	Rodentia	Cricetidae	0.68	0.16	57.38	0.05	0.08	1.53	1.67	-0.81	42.00	61.00	Lüke et al 2011	Gómez Montoto et al 2011b	Gómez Montoto et al 2011a
Microtus_gerbei	Rodentia	Cricetidae	0.65	0.17	58.06						44.00	62.00	Lüke et al 2011		
Mus_cookii	Rodentia	Muridae	0.68	0.13	50.79	0.03	0.07	2.00	1.37	-0.52	44.00	63.00	FJ411386	Lüke et al 2014	Gómez Montoto et al 2011
Mus_famulus	Rodentia	Muridae	0.74	0.12	52.38	0.03	0.07	1.97	1.44	-1.28	44.00	63.00	FJ411388	Lüke et al 2014	Gómez Montoto et al 2011
Mus_macedonicus	Rodentia	Muridae	0.74	0.12	50.79	0.03	0.07	2.06	1.30	-0.53	44.00	63.00	FJ411391	Lüke et al 2014	Gómez Montoto et al 2011
Mus_musculus_bactrianus	Rodentia	Muridae	0.76	0.12	50.79	0.03	0.06	2.22	1.26	-0.76	44.00	63.00	FJ411384	Lüke et al 2014	Gómez Montoto et al 2011
Mus_musculus_castaneus	Rodentia	Muridae	0.76	0.12	50.79	0.03	0.06	2.26	1.27	-1.12	44.00	63.00	FJ411385	Lüke et al 2014	Gómez Montoto et al 2011
Mus_musculus_domesticus	Rodentia	Muridae	0.74	0.12	50.79	0.03	0.06	2.03	1.34	-0.96	44.00	63.00	FJ411387	Lüke et al 2014	Gómez Montoto et al 2011
Mus_musculus_musculus	Rodentia	Muridae	0.76	0.12	50.79	0.03	0.08	2.52	1.34	-0.86	44.00	63.00	FJ411383	Gómez Montoto et al 2011b	Gómez Montoto et al 2011a
Mus_pahari	Rodentia	Muridae	0.68	0.13	50.79	0.04	0.07	1.94	1.52	-0.89	44.00	63.00	FJ411389	Lüke et al 2014	Gómez Montoto et al 2011a
Mus_spicilegus	Rodentia	Muridae	0.71	0.13	52.38	0.03	0.08	2.47	1.26	-0.37	44.00	63.00	FJ411392	Gómez Montoto et al 2011b	Gómez Montoto et al 2011a
Mus_spretus	Rodentia	Muridae	0.74	0.12	52.38	0.04	0.08	2.15	1.26	-0.52	44.00	63.00	FJ411390	Gómez Montoto et al 2011b	Gómez Montoto et al 2011a
Myotis_lucifugus	Chiroptera	Vespertilionidae	0.81	0.28	62.26	0.04	0.08	2.39	0.83	-0.97	36.00	53.00	BK006495	Cummins & Woodall 2008	Hosken 1997
Otolemur_garnettii	Primates	Lemuridae	2.08	0.27	53.23						40.00	62.00	BK006492		
Pan_paniscus	Primates	Pongidae	1.75	0.30	48.15		0.07		4.59	2.13	48.00	54.00	X71334	Anderson et al 2005	Dixson & Anderson 2004
Pan_troglodytes	Primates	Pongidae	1.65	0.31	48.15		0.08		4.65	2.07	48.00	54.00	NM_001009084	Cummins & Woodall 1985	Kenagy & Trombulak 1986
Papio_anubis	Primates	Cercopithecidae	1.41	0.26	58.18		0.06		4.42	1.89	45.00	55.00	101009198.00	Anderson et al 2005	Dixson & Anderson 2004
Phodopus_campbelli	Rodentia	Cricetidae	0.64	0.14	50.00	0.03	0.06	2.46	1.69	0.29	40.00	62.00	Lüke et al 2011	Lüke et al 2014	
Phodopus_roborovski	Rodentia	Cricetidae	0.67	0.19	50.79	0.03	0.06	2.21	1.41	0.03	40.00	63.00	Lüke et al 2011	Lüke et al 2014	Ramm et al 2008
Phodopus_sungorus	Rodentia	Cricetidae	0.64	0.14	49.21	0.02	0.06	2.48	1.66	0.02	40.00	63.00	Lüke et al 2011	Gage & Freckleton 2003; Gage 1998	Hoffman 1979
Pitimys_duodecimcostatus	Rodentia	Cricetidae	0.65	0.17	58.06	0.05	0.08	1.59	1.44	-1.08	44.00	62.00	Lüke et al 2011	Gómez Montoto et al 2011b	Gómez Montoto et al 2011a
Pitimys_lusitanicus	Rodentia	Cricetidae	0.65	0.16	58.06	0.04	0.07	1.84	1.27	-0.94	44.00	62.00	Lüke et al 2011	Gómez Montoto et al 2011b	Gómez Montoto et al 2011a
Pongo_abelii	Primates	Pongidae	1.66	0.28	44.44						48.00	54.00	100446402.00		
Pongo_pygmaeus	Primates	Pongidae	1.66	0.28	44.44		0.08		4.87	1.55	48.00	54.00	X71337	Cummins & Woodall 1985	Kenagy & Trombulak 1986
Rattus_fuscipes	Rodentia	Muridae	0.73	0.14	49.18		0.07		2.04	0.63	44.00	61.00	AF268201	Cummins & Woodall 1985	Breed & Taylor 2000
Rattus_norvegicus	Rodentia	Muridae	0.72	0.17	48.33		0.06		2.58	0.49	44.00	60.00	NM_012873.1	Cummins & Woodall 1985	Kenagy & Trombulak 1986; Wu et al 2010
Rattus_tunneyi	Rodentia	Muridae	0.72	0.17	50.82		0.11		2.39	0.69	44.00	61.00	AF268199	Olds et al	Breed 1997
Saimiri_boliviensis	Primates	Cebidae	0.96	0.24	60.71						48.00	56.00	101051182.00		
Semnopithecus_entellus	Primates	Cercopithecidae	0.98	0.25	56.36				4.27	1.05	48.00	55.00	AF195642		Harrison & Lewis 1986
Sigmodon_hispidus	Rodentia	Cricetidae	0.50	0.14	50.00	0.04	0.07	1.91	2.35	0.24	42.00	62.00	EU980396	Cummins & Woodall 1985	Kenagy & Trombulak 1986
Sus_scrofa	Artiodactyla	Suidae	1.55	0.24	60.42	0.09	0.16	1.70	4.60	2.11	44.00	48.00	NM_214252	Cummins & Woodall 1985; Gage 1998	Almeida et al 2006

References

Almeida FF, Leal MC, França LR (2006) Biol Reprod 75:792–799. **Cummins JM, Woodall PF** (1985) J Reprod Fertil 75:153–175. **Dixson AF, Anderson MJ** (2004) Physiol Behav 83:361–371. **Gage MJG** (1998) Proc Roy Soc Lond B Bio 265:97–103. **Gage MJG, Freckleton R** (2003) Proc Roy Soc Lond B 270:625–632. **L Gómez-Montoto, ERS. Roldan, M Gomendio**, unpublished data. **Gómez Montoto L, Magaña C, Tourmente M, Martín-Coello J, Crespo C et al.** (2011) PLoS ONE 6: e18173. **Gómez Montoto L, Varea Sánchez M, Tourmente M, Martín-Coello J, Luque-Larena JJ, Gomendio M, Roldan ERS** (2011b) Reproduction 142:819–830. **Harrison RM, Lewis RW** (1986) in Dukelow WR, Erwin J (eds): Comparative Primate Biology. New York, Liss, vol 3, pp 101–148. **Kenagy GJ, Trombulak** C (1986) J Mammal 67:1–22. **Lüke L, Vicens A, Serra F, Luque Larena JJ, Dopazo H, Roldan ERS, Gomendio M** (2011) PLoS ONE, 6:e29247. **Lüke L, Vicens A, Tourmente M, Roldan ERS** (2014) Biol Reprod 90:67. **Ramm SA, Oliver PL, Ponting CP, Stockley P, Emes RD** (2008) Mol Biol Evol 25: 207. **Ramm SA, Parker GA, Stockley** P (2005) Proc R Soc B, 272:949–955. **Slott PA, Liu MH, Tavoloni N** (1990) Gastroenterology 99:466–477. **Wu PF, Chiang TA, Chen MT, Lee CP, Chen PH, Ko AM, Yang KJ, Chang PY, Ke DS, Ko YC** (2010) J Hazard Mater 178:541-546.
