# Supplementary material for: Selective constraints on protamine 2 in primates and rodents
Source: BMC Evol Biol. 2016 Jan 22;16:21. doi: 10.1186/s12862-016-0588-1 (PMC4724148; doi:10.1186/s12862-016-0588-1)
Supplement: Additional file 2: Figure S1. — Phylogenetic tree constructed as consensus of phylogenetic data available in the literature. (PDF 650 kb) [file 12862_2016_588_MOESM2_ESM.pdf]

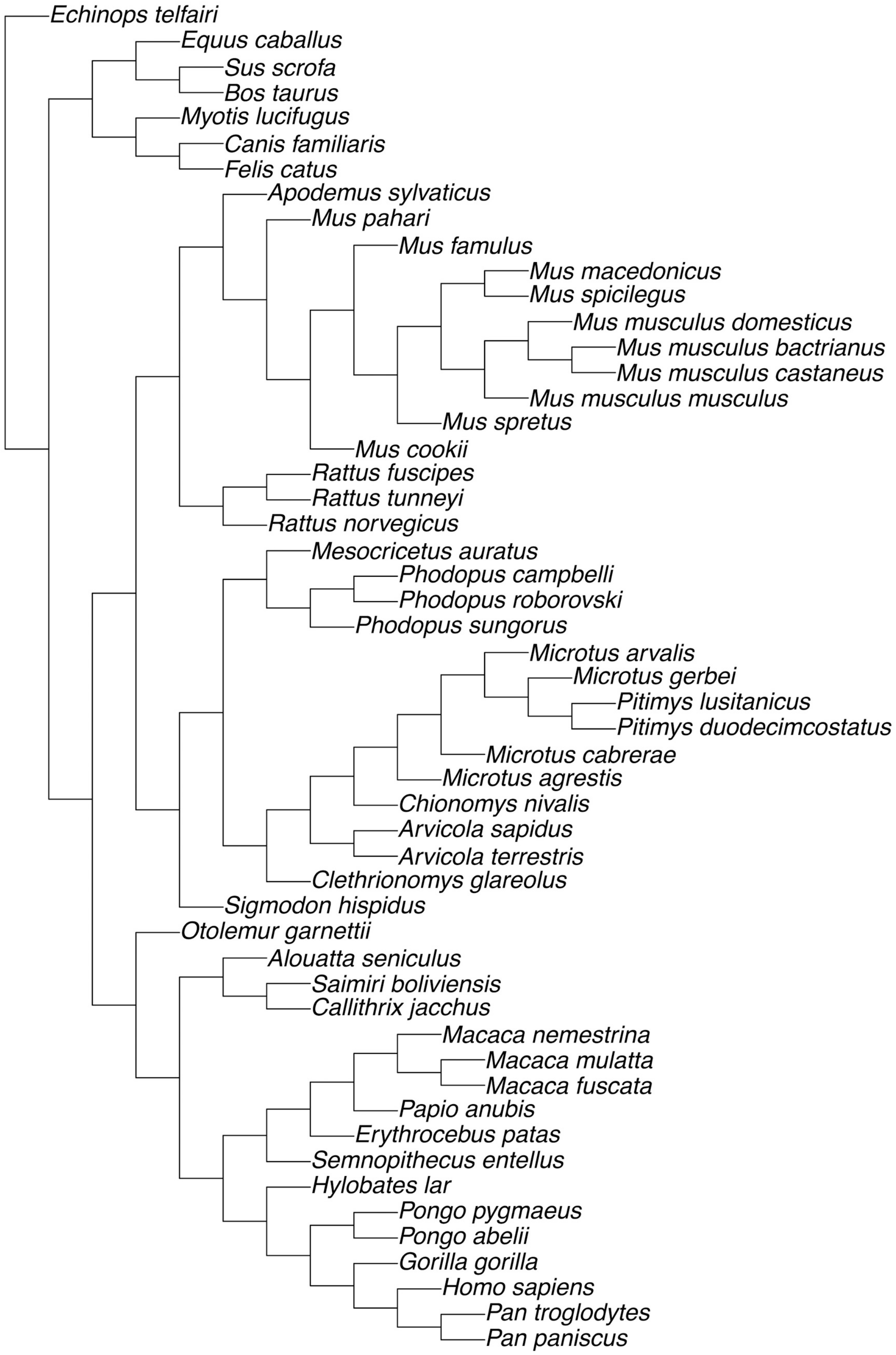

Figure S1. Phylogenetic tree constructed as consensus of phylogenetic data available in the literature .

References

Adkins RM, Walton AH, Honeycutt RL. Higher-level systematics of rodents and divergence time estimates based on two congruent nuclear genes. Mol. Phylogenet. Evol. 2003;26:409-420.

Almeida FC, Bonvicino CR, Cordeiro-Estrela P. Phylogeny and temporal diversification of Calomys (Rodentia, Sigmodontinae): Implications for the biogeography of an endemic genus of the open/dry biomes of South America. Mol. Phylogenet. Evol. 2007;42:449-466.

Alvarez A, Perez SI, Verzi DH. Ecological and phylogenetic influence on mandible shape variation of South American caviomorph rodents (Rodentia: Hystricomorpha). Biol. J. Linn. Soc. 2011;102:828-837.

Baena A, Mootnick AR, Falvo JV, Tsytskova AV, Ligeiro F, Diop OM, Brieva C, Gagneux P, O'Brien SJ, Ryder OA, Goldfeld AE: Primate TNF promoters reveal markers of phylogeny and evolution of innate immunity. PLoS One. 2005;2:e621.

Beck RM, Bininda-Emonds OR, Cardillo M, Liu FR, Purvis A. A higher-level MRP supertree of placental mammals. BMC Evol. Biol. 2006;6:93-107.

Bininda-Emonds ORP, Cardillo M, Jones KE, MacPhee RDE, Beck RMD, Grenyer R, Price SA, Vos RA, Gittleman JL, Purvis A. The delayed rise of present-day mammals. Nature. 2007;446:507-512.

Böhm M, Mayhew PJ. Historical biogeography and the evolution of the latitudinal gradient of species richness in the Papionini (Primata: Cercopithecidae). Biol. J. Linn. Soc. Lond. 2005;85:235-246.

Borges BN, Paiva TS, Harada ML. Evolution of the SEC1 gene in New World monkey lineages (Primates, Platyrrhini). Genet. Mol. Res. 2008;7:663-678.

Conroy CJ, Cook JA. Molecular systematics of a holartic rodent (Microtus: Muridae). J. Mammal. 2000;81:344-359.

Flynn JJ, Finarelli JA, Zehr S, Hsu J, Nedbal MA. Molecular phylogeny of the Carnivora (Mammalia): assessing the impact of increased sampling on resolving enigmaticrelationships. Syst. Biol. 2005;54:317-337.

Gomendio M, Tourmente M, Roldan ERS () Why mammalian lineages respond differently to sexual selection: metabolic rate constrains the evolution of sperm size. Proc. Roy. Soc. Lond. B. 2011;278:3135-3141.

Gómez Montoto L, Magaña C, Tourmente M, Martín-Coello J, Crespo C, Luque-Larena JJ, Gomendio M, Roldan ERS. Sperm competition, sperm numbers and sperm quality in muroid rodents. PLoS One. 2011;6:e18173.

Johnson WE, Eizirik E, Pecon-Slattery J, Murphy WJ, Antunes A, Teeling E, O'Brien SJ. The late Miocene radiation of modern Felidae: a genetic assessment. Science. 2006;311:73-77.

Kjer KM, Honeycutt RL () Site specific rates of mitochondrial genomes and the phylogeny of eutheria. BMC Evol. Biol. 2007;7:8-17.

Lecompte E, Aplin K, Denys C, Catzeflis F, Chades M, Chevret P. Phylogeny and biogeography of African Murinae based on mitochondrial and nuclear gene sequences,with a new tribal classification of the subfamily. BMC Evol. Biol. 2008;8:199-220.

Liu X, Wei F, Li M, Jiang X, Feng Z, Hu J. Molecular phylogeny and taxonomy of wood mice (genus Apodemus Kaup, 1829) based on complete mtDNA cytochrome bsequences, with emphasis on Chinese species. Mol. Phylogenet. Evol. 2004;33:1-15.

Menzies JI. A systematic revision of Melomys (Rodentia: Muridae) of New Guinea. Aust. J.

Zool. 1996;44:367-426.

Michaux J, Chevret P, Renaud S. Morphological diversity of Old World rats and mice (Rodentia, Muridae) mandible in relation with phylogeny and adaptation. J. Zool. Syst. Evol. Res. 2007;45:263-279.

Poux C, Douzery EJ. Primate phylogeny, evolutionary rate variations, and divergence times: a contribution from the nuclear gene IRBP. Am. J. Phys. Anthropol. 2004;124:1-16.

Prasad AB, Allard MW, Program NCS, Green ED. Confirming the phylogeny of mammals by use of large comparative sequence data sets. Mol. Biol. Evol. 2008;25:1795-1808.

Prothero DR, Foss SE. The Evolution of Artiodactyls (JHU Press, Baltimore, USA); 2007.

Sears KE, Finarelli JA, Flynn JJ, Wyss AR. Estimating body mass in New World monkeys (Platyrrhini, Primates) with a consideration of the Miocene platyrrhine, Chilecebus carrascoensis. Am. Mus. Novitates. 2008;3167:1-29.

Seddon JM, Baverstock PR. Evolutionary Lineages of RT1.Ba in the Australian Rattus. Mol. Biol. Evol. 2000;17:768-772.

Springer MS, Murphy WJ. Mammalian evolution and biomedicine: new views from phylogeny. Biol. Rev. 2007;82:375-392.

Steiper ME, Ruvolo M. New World monkey phylogeny based on X-linked G6PDDNA sequences. Mol. Phylogenet. Evol. 2003;27:121-130.

Steppan SJ, Adkins RM, Anderson J. Phylogeny and divergence-date estimates of rapid radiations in Muroid rodents based on multiple nuclear genes. Syst. Biol. 2004;53:533-553.

Steppan SJ, Adkins RM, Spinks PQ, Hale C. Multigene phylogeny of the Old World mice, Murinae, reveals distinct geographic lineages and the declining utility of mitochondrial genes compared to nuclear genes. Mol. Phylogenet. Evol. 2005;37:370-388.

Swann CA, Cooper SJ, Breed WG. Molecular evolution of the carboxy terminal region of the zona pellucida 3 glycoprotein in murine rodents. Reproduction. 2007;133:697-708.

Valdespino C. Physiological constraints and latitudinal breeding season in the Canidae. Physiol. Biochem. Zool. 2007;80:580-591.

Xing J, Wang H, Han K, Ray DA, Huang CH, Chemnick LG, Stewart CB, Disotell TR, Ryder OA, Batzer MA. A mobile element based phylogeny of Old World monkeys. Mol. Phylogenet. Evol. 2005;37:872-880.

Yoder AD, Yang Z. Divergence dates for Malagasy lemurs estimated from multiplegene loci: geological and evolutionary context. Mol. Ecol. 2004;13:757-773.
