# Supplementary figures and images for: Selective constraints on protamine 2 in primates and rodents
Source: BMC Evol Biol. 2016 Jan 22;16:21. doi: 10.1186/s12862-016-0588-1 (PMC4724148; doi:10.1186/s12862-016-0588-1)

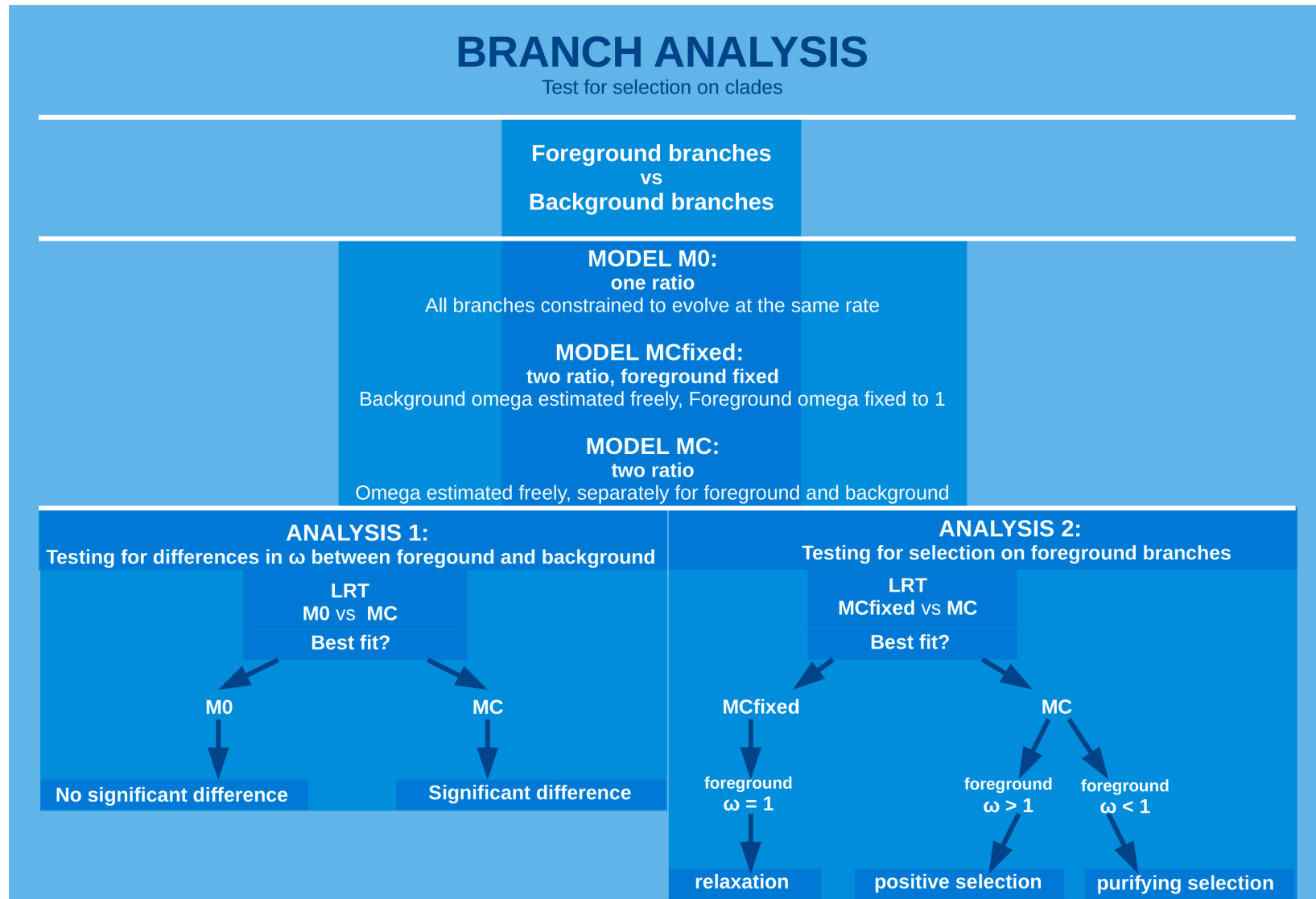

Supplement: Additional file 3: Figure S2. — Graphical representation of the analyses carried out in this study. (PDF 1607 kb) [file 12862_2016_588_MOESM3_ESM.pdf]
